# Diagnostic Challenges in Acute Leukemia: From Dental Pain to Catastrophic Intracerebral Hemorrhage

**DOI:** 10.3390/hematolrep17040036

**Published:** 2025-07-23

**Authors:** Anatoli Pinchuk, Stefan P. Roch, Christian Mawrin, Daniel Behme, Klaus-Peter Stein, Belal Neyazi, Martin Mikusko, Ibrahim Erol Sandalcioglu, Ali Rashidi

**Affiliations:** 1 Department of Neurosurgery, Otto von Guericke University, 39120 Magdeburg, Germany; anatoli.pinchuk@med.ovgu.de (A.P.); stefan.roch@med.ovgu.de (S.P.R.); klaus-peter.stein@med.ovgu.de (K.-P.S.); belal.neyazi@med.ovgu.de (B.N.); erol.sandalcioglu@med.ovgu.de (I.E.S.); 2 Department of Neuropathology, Otto von Guericke Unviersity, 39120 Magdeburg, Germany; christian.mawrin@med.ovgu.de; 3 Department of Neuroradiology, Otto von Guericke University, 39120 Magdeburg, Germany; daniel.behme@med.ovgu.de; 4 Department of Hematology, Oncology and Cell Therapy, Otto von Guericke University, 39120 Magdeburg, Germany; martin.mikusko@med.ovgu.de

**Keywords:** acute leukemia, toothache, fatal hemorrhage, intracerebral hemorrhage, disseminated intravascular coagulation, thrombocytopenia, case report

## Abstract

**Background and Clinical significance**: Acute leukemias are neoplasms of the hematopoietic system that are caused by the extensive proliferation of immature precursor cells (‘blasts’), mainly in the bone marrow. They frequently manifest with vague and non-specific clinical symptoms, making early diagnosis particularly challenging. **Case Presentation**: This case report describes the clinical course of a female patient who initially sought dental care due to a persistent toothache—an atypical and misleading symptom. Subsequent investigations revealed a diagnosis of acute leukemia. Although the malignancy was identified promptly and the appropriate therapeutic measures were initiated, the disease progressed with alarming rapidity. The patient ultimately developed a massive intracerebral hemorrhage—a devastating complication likely related to leukemia-associated coagulopathy. Despite emergent neurosurgical intervention, the hemorrhage proved fatal. **Conclusions**: This case highlights the critical need for heightened clinical suspicion in the presence of unusual symptoms and illustrates the complex interplay between hematologic malignancies and coagulopathic complications.

## 1. Introduction

Acute leukemias are clonal neoplasms of the hematopoietic system, in which immature precursor cells (‘blasts’) proliferate extensively in the bone marrow and can frequently be found in the peripheral blood. This pathological proliferation leads to the blockage of normal differentiation and thus to rapid bone marrow failure, with anemia, thrombocytopenia, and neutropenia. The aforementioned findings result in significant clinical manifestations and potentially lethal complications, such as severe infections; an increased tendency to bleed even with the onset of disseminated intravascular coagulation and hyperfibrinolysis; and leukocytosis, with the possibility of leukostasis, resulting in hypoxia-associated complications and neurological symptoms.

Acute leukemias can be categorized into two major forms—acute lymphoblastic leukemia (ALL) and acute myeloid leukemia (AML). The initial clinical presentation of both diseases may be characterized by non-specific general symptoms, including fatigue, fever, and bleeding. In the context of AML, the potential for specific oral manifestations, such as gingival hyperplasia and bleeding, is a significant consideration [1,2,3].

Oral complications, such as gingival hyperplasia, are frequently observed in FAB subtypes M5 (acute monocytic leukemia) and M4 (acute myelomonocytic leukemia) [4,5]. Gingival hyperplasia is also likely in the subleukemic phase [6]. A recent meta-analysis showed a high prevalence of oral signs of disease in a pediatric population suffering from acute leukemia, with caries reported in 81%, oral gingivitis in 73%, and oral mucositis in 50% of patients during treatment [7]. In the case of acute promyelocytic leukemia (APL or AML M3), a distinct form of acute myeloid leukemia (AML), oral symptoms can be particularly pronounced, with patients experiencing spontaneous gingival bleeding and swelling [8]. The presence of toothache in these patients may be misdiagnosed as a common dental complaint, delaying the recognition of underlying hematological conditions.

Intracerebral hemorrhage (ICH) is a serious complication associated with acute leukemia, particularly in patients with hyperleukocytosis and thrombocytopenia [9,10]. Among all hematologic malignancies, ICH occurs most frequently in patients suffering from AML [11,12]. ICH may occur as a result of leukostasis, in which the high number of leukemic cells obstructs small blood vessels, leading to vascular damage, and hemorrhage ensues [13]. Studies have shown that ICH is a leading cause of mortality in patients with acute leukemia, with an incidence of up to 6% in AML patients [9,10]. The risk factors for ICH include severe thrombocytopenia, disseminated intravascular coagulation (DIC), and the presence of leukemic cells in the central nervous system [9,12]. Early diagnosis and treatment are crucial for successfully managing such cases, as the presence of oral symptoms can often serve as an indicator of hemorrhagic complications in AML [14,15]. In general, a diagnosis of acute leukemia demands prompt medical intervention in order to effectively manage its aggressive nature and associated complications [12,16].

The connection between toothache and ICH in acute leukemia underscores the importance of thorough clinical evaluations in patients presenting with inapparent oral symptoms and may be the first lead to revealing severe underlying morbidity and mortality. Dentists and healthcare providers should be vigilant in recognizing these signs as potential indicators of serious underlying conditions, facilitating timely diagnosis and intervention. Early detection of leukemia contributes significantly to improving its prognosis and reducing the risk of severe complications such as ICH [3,12].

In summary, acute leukemia can manifest with initially inapparent symptoms such as toothache, disguising the underlying signs of a hemorrhage. Although recognizing these inapparent and unspecific symptoms proves difficult, it may, however, serve as a critical indicator for an early diagnosis. Understanding the relationship between these symptoms and the underlying hematological disorder is essential for healthcare providers to ensure prompt and effective treatment for affected patients.

We present a case illustrating the diagnostic challenge of a patient with acute leukemia which took a rapidly fatal turn.

## 2. The Case Report

This case involves a 55-year-old woman with a history of essential hypertension. The patient had been experiencing toothache, fever, and general weakness for several days before presenting to a private practice dentist on 24 February 2021. Following examination, intraoral bloody swelling was noted, alongside an overall exsiccated general condition. She was subsequently referred to the oral and maxillofacial surgery and neurology departments for further assessment and management. Approximately a week prior, she presented to her primary care physician with general fatigue; however, since the physical examination was inconspicuous, no laboratory examinations were undertaken, and follow-up was recommended.

**Figure 1:**
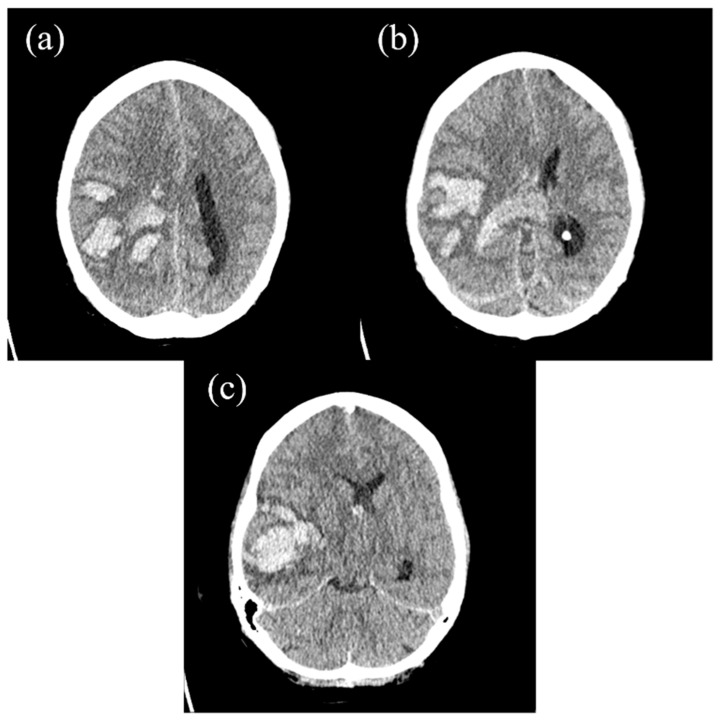
(**a**–**c**) The axial cranial CT scan showing a massive, multilocular ICH in the right hemisphere, causing a significant midline shift due to the local mass effect and intraventricular hemorrhage.

After presentation at the emergency department, the patient turned soporous, accompanied by circulatory dysregulation. Intubation and mechanical ventilation became necessary. A cranial CT scan was prompted and revealed multilocular intracerebral hemorrhages on the right side (Figure 1). CT angiography of the head and neck showed no vascular abnormalities. The standard management for ICH was instantly initiated, including blood pressure regulation, hypertonic fluid administration to reduce cerebral edema, and seizure prophylaxis. The patient was subsequently taken to the operating room for decompressive hemicraniectomy and hematoma evacuation.

Subsequent to surgery, the patient was transferred to the neurosurgical intensive care unit under sedation and mechanical ventilation. A neurological examination revealed anisocoria in favor of the right pupil and extensor posturing.

The initial laboratory evaluation showed significantly elevated levels of C-reactive protein (CRP), at 123.40 mg/L; aspartate aminotransferase (ASAT), at 1.32 µmol/s; and gamma-glutamyl transferase (GGT), at 0.980 µmol/s/L. Coagulation studies revealed a Quick value of 54% and an international normalized ratio (INR) of 1.39. A complete blood count (CBC) indicated microcytic anemia with a hemoglobin level of 6.9 mmol/L, thrombocytopenia with a platelet count of 35,000/μL, and a markedly elevated leukocyte count at 218,000/μL. Strong suspicion of acute myeloid leukemia (AML) was raised.

A morphological assessment of her blood smear revealed a significant increase in large cells (69%), some of which exhibited polymorphic nuclei and loose nuclear chromatin, small nucleoli, and a variable width of blue-gray to basophilic cytoplasm, frequently accompanied by vacuoles. Consequently, the cytomorphological picture was most consistent with acute leukemia, most likely acute myeloid leukemia. This finding was subsequently confirmed through a flow cytometric analysis. In this particular case, an immature myeloid cell population (88.2% of all CD45-positive cells) was identified, exhibiting specific expressions of CD13/CD15partial/CD33/CD34; partial/CD38/CD45; medium-high/CD64; low/CD65; and partial/CD117/CD123/CD200/HLA-DR/MPO but not CD2/cCD3/CD3/CD4/CD7/CD14/CD16/CD19/CD20/cCD22/CD56/cCD79a/CD135/NG2/cIgM/cTdT.

**Figure 2:**
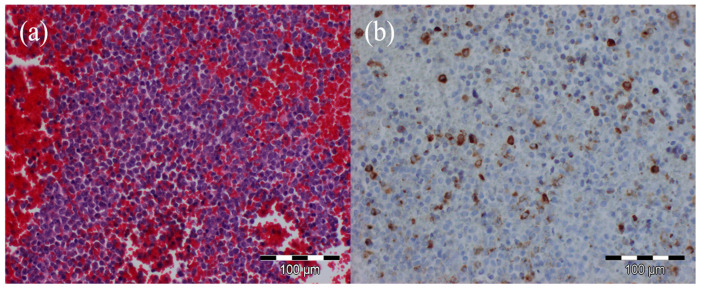
(**a**) Peripheral blood smear stained with hematoxylin and eosin showing numerous blast cells. (**b**) Peripheral blood smear stained for Myeloperoxidase.

Further laboratory and microscopy studies of brain tissue obtained during the surgery demonstrated the marked proliferation of myeloid cells, with limited mitotic activity. Myeloperoxidase staining was positive (Figure 2), and specific staining for CD68 indicated significant macrophage involvement. The proliferation marker MiB1 was positive in approximately 15–20% of cells.

**Figure 3:**
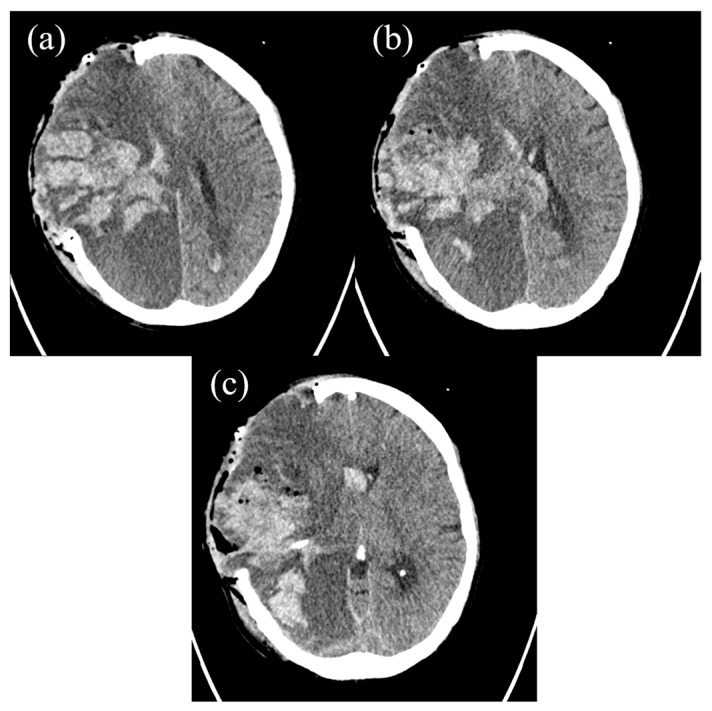
(**a**–**c**) The postoperative axial cranial CT scan revealing further ICH in the right frontal and parietal lobes.

Given the severe coagulopathy and thrombocytopenia, substitution therapy with prothrombin complex concentrate (PCC), packed platelets, and red blood cells was administered. A postoperative cranial CT scan revealed further ICH in the right frontal and parietal lobes (Figure 3).

The peripheral blood smear analysis confirmed 69% blasts (Figure 2). The microscopy findings, in conjunction with the immunohistochemical staining results, solidified the diagnosis of AML. Following consultation with hematology and oncology specialists, the laboratory and clinical findings were deemed consistent with acute myeloid leukemia.

Despite intensive medical care, the patient’s neurological status worsened. Follow-up cranial CT scans demonstrated extensive bilateral infarction, and her brainstem reflexes diminished progressively. Given the dismal prognosis, the patient’s family asked for intensive medical care to be terminated. The patient passed away shortly after.

## 3. Discussion

The diagnosis and treatment of acute myeloid or lymphoblastic leukemia present significant challenges, particularly when patients exhibit atypical and inapparent symptoms such as a toothache. This symptom may be missed by primary healthcare providers, as it may be mistaken for a common dental issue rather than an indicator of an underlying, severe hematological malignancy. Infiltration of the gingiva by leukemic cells can mimic conditions like gingivitis or periodontal disease, leading to delays in diagnosis and treatment [14]. Such misdiagnosis can have dire consequences, as timely intervention is crucial for improving the patient outcomes in acute leukemia.

If acute leukemia is left untreated or misdiagnosed, life-threatening conditions such as severe infections, severe bleeding, and hypoxia-associated complications can develop. The majority of untreated patients succumb to AML within one year [17]. Moreover, the less common but life-threatening nature of the hemorrhagic complications associated with DIC in leukemia cannot be overstated. Patients with AML are particularly vulnerable to hemorrhagic events due to thrombocytopenia, DIC, and a fragile vasculature resulting from leukemic infiltration [9,10]. Under these conditions, even spontaneous systemic hemorrhages may occur without any trauma; thereby, the risk of spontaneous ICH is markedly increased [10].

In cases with significant ICH, aggressive transfusion, coagulation factor substitution, and immediate surgical intervention are often required to manage acute cases. Despite all efforts, these measures may prove insufficient, as evidenced by cases in which patients continue to experience severe complications [18,19]. Patients that develop ICH often carry a poor prognosis with high mortality rates, as was experienced in the case presented [9].

The pathogenesis of ICH in hyperleukocytosis is multifactorial: it can involve mechanical obstruction of the small vessels, which equates to greater endothelial adhesion and thus greater invasion [20]. In addition, hyperleukocytosis causes hyperviscosity and leukostasis, leading to hypoxic vasodilation, increased vascular permeability, and rupture of the small cerebral vessels [21]. Other risk factors include DIC, sepsis, and vessel wall abnormalities [22]. The patient had several risk factors contributing to ICH, as listed in other studies, namely hypertension, marked leukocytosis, and thrombocytopenia. The mortality rate in such cases has been reported to be over 50%, and death often occurs in the first 48 h [9]. In a systematic review, Yu et al. described leukocytosis at the baseline as significantly correlated with poor long-term functional outcomes in patients with ICH and higher mortality rates [23].

The PATCH study found that platelet transfusions were associated with a significant increase in adverse events. However, the guidelines recommend platelet transfusions for patients undergoing neurosurgical procedures. In our case, the patient was actively bleeding with a platelet count of <50,000/L, warranting transfusion [24]. The treatment for hyperleukocytosis is disputed; however, options include cytoreduction, either through hydroxyurea or induction chemotherapy, as well as leukapharesis [25].

Previous case reports have described similar patient fates. Similarly to our case, Suárez-Cuenca et al. described a case of a 24 y old male presenting to a dentist with toothache and oral petechiae, as well as mandibular swelling and submandibular ecchymosis. No laboratory examinations were performed. Following two tooth extractions, the patient experienced intractable pain and bleeding warranting hospital treatment; APL was then diagnosed. Following seizures, ICH was found, and the patient also succumbed to hemorrhagic complications, as in our case [8]. Another case report by Koi et al. described a 43 y old male that was diagnosed with APL following oral bleeding. Coincidentally, during the treatment course, ICH was diagnosed in cranial MRI for the assessment of CNS involvement. The patient presented no neurological signs and was conservatively treated with platelet transfusions; the overall outcome was reported to be APL remission, with no neurological symptoms [26].

In summary, the interplay of atypical symptoms leading to a delayed diagnosis or misdiagnosis and the risk of hemorrhagic complications underscores the importance of vigilance among healthcare providers, as it requires aggressive management strategies. Dentists and hematologists must work collaboratively to ensure that oral manifestations of leukemia are recognized early, facilitating prompt diagnosis and treatment [3,14]. This multidisciplinary approach is essential for improving the management of acute leukemia and mitigating the risks associated with its complications.

## 4. Conclusions

Healthcare professionals ought to consider hematologic malignancies in the differential diagnosis of unexplained toothaches with signs of hemorrhage, especially when accompanied by systemic symptoms or abnormal laboratory findings. This case emphasizes the critical importance of early diagnosis and multidisciplinary management to addressing the life-threatening complications of acute leukemia.

## Data Availability

The original contributions presented in this study are included in the article. Further inquiries can be directed to the corresponding author.

## Abbreviations

The following abbreviations are used in this manuscript:

AML	Acute Myeloid Leukemia
ALL	Acute Lymphoblastic Leukemia
DIC	Disseminated Intravascular Coagulopathy
APL	Acute Promyelocytic Leukemia
ICH	Intracerebral Hemorrhage
CT	Computed Tomography
CRP	C-Reactive Protein
ASAT	Aspartate Aminotransferase
GGT	Gamma-Glutamyl Transferase
INR	International Normalized Ratio
CBC	Complete Blood Count
CD	Cluster of Differentiation
PCC	Prothrombin Complex Concentrate
L	Liter
